# Postoperative Outcomes of Masseteric Nerve Transposition versus Cross-Facial Nerve Graft in Facial Reanimation: A Systematic Review and Meta-Analysis

**DOI:** 10.1055/a-2707-0408

**Published:** 2026-01-30

**Authors:** Indri Lakshmi Putri, Klarina Elsa Siti Sarah, Imaniar Fitri Aisyah, Rachmaniar Pramanasari, Citrawati Dyah Kencono Wungu

**Affiliations:** 1 Department of Plastic Reconstructive and Aesthetic Surgery, Faculty of Medicine, Airlangga University, Surabaya, Indonesia; 2 Plastic Reconstructive and Aesthetic Surgery Unit, Airlangga University Hospital, Surabaya, Indonesia; 3 Department of Mechanical Engineering, Institut Teknologi Sepuluh Nopember, Surabaya, Indonesia; 4 Department of Physiology and Medical Biochemistry, Faculty of Medicine, Airlangga University, Surabaya, Indonesia; 5 Institute of Tropical Disease, Airlangga University, Surabaya, Indonesia

**Keywords:** facial nerve paralysis, facial reanimation, masseteric nerve transposition, cross-facial nerve graft, postoperative outcome

## Abstract

Facial reanimation surgery offers various approaches, with the choice of method influenced by numerous factors. Masseteric nerve transposition (MNT) has become a preferred neural source, often compared with cross-face nerve graft (CFNG) for its respective advantages. This systematic review and meta-analysis aim to compare postoperative outcomes between MNT and CFNG in facial reanimation surgery for patients with facial nerve paralysis sequelae.

A comprehensive electronic search was conducted using databases such as PubMed/Medline, Scopus, ScienceDirect, EBSCO, Web of Science, and Cochrane Library, along with gray literature sources like Scopus Preprints and MedRxiv. Statistical analyses were performed using Review Manager version 5.4.1, with the quality of included studies assessed using the Newcastle–Ottawa Scale.

Results showed that MNT provided statistically significant improvements in commissural excursion (CE; mean difference [MD] = 2.36 mm) and commissural contraction velocity (CCV; MD = 7.01 mm/s) compared with CFNG. Additionally, MNT had a higher recovery percentage for CE (MD = 26.86%) and CCV (MD = 13.00%). Superior outcomes were also noted for static and dynamic smile symmetry, quality of life, and patient satisfaction.

This meta-analysis underscores the advantages of MNT over CFNG in the majority of parameters in the analysis, at once highlighting the need for further research with larger sample sizes for more precise comparisons.

## Introduction

Reanimation of the paralyzed face depends on several key factors, with denervation time being one of the most critical determinants. 1 2 Prolonged denervation is associated with a higher likelihood of unsuccessful outcomes. 3 4 For instance, in patients with short-term facial paralysis (ranging from 3 months to 2 years), the facial musculature remains viable, making nerve transposition a favorable option. 5 6 In contrast, for long-standing paralysis where the facial muscles are no longer viable, a new muscle unit must be introduced to restore motion. 1 7

There is considerable variation in expert recommendations regarding the optimal nerve source for reanimation. 8 Common neurotization options include the cross-facial nerve graft (CFNG), as well as the masseteric, hypoglossal, accessory, and phrenic nerves. 6 9 10 11 Historically, the CFNG has been considered one of the most effective choices for facial reanimation, primarily due to its ability to facilitate spontaneous contraction. 11 12 13 14 However, the procedure can be limited by factors such as reduced oral commissure excursion, which results from a lower axonal count, a longer nerve regeneration distance, and a higher incidence of donor site morbidity. 15

Masseteric nerve transposition (MNT) has recently become one of the most frequently used neural sources for enhancing facial function due to its high axonal density 8 16 and favorable anatomical position. 11 13 17 18 It has emerged as a standard option for patients who are not suitable candidates for CFNG. 19 20 MNT offers several advantages, including improved muscle contraction strength and an expanded window for restoring facial nerve function, with minimal impairment at the donor site. 20 21 The key benefits of MNT include reduced morbidity, 5 proximity to the facial nerve, a robust motor impulse, reliability, and rapid reinnervation, making it a viable option for most patients. 16 18 22 23 This technique is applicable to both chronic and acute facial palsy (within 24 months). 7 24

Previous studies comparing MNT and CFNG have often been limited in scope, focusing on the specific muscle used for reanimation, employing a single assessment tool, or constrained by the nature of the cases studied. Therefore, it is essential to determine which reanimation technique provides the best outcomes across different contexts. This study aims to offer a comprehensive comparison of MNT and CFNG, with an emphasis on specific functional outcomes in both adult and pediatric populations. The comparison includes multiple etiologies, regardless of which muscle is involved, and covers cases of complete or incomplete paralysis, as well as unilateral or bilateral facial palsy.

Facial reanimation surgery aims to restore both static and dynamic facial symmetry, achieve oral competence, enable eye closure, 21 25 promote voluntary facial movement, and facilitate effortless, spontaneous expression without synkinesis, all while minimizing functional loss at the donor site. Clinical experience and research suggest that asymmetry of the smile is often the primary concern for individuals with facial paralysis, 26 as facial symmetry plays a crucial role in perceived attractiveness. 27 28 29 The effectiveness of these interventions can be evaluated using a range of techniques, metrics, and methodologies. 24 25 30 31 32

## Methods

### Search Strategy

A comprehensive database search was conducted by the first author in April 2024 across Scopus, PubMed, ScienceDirect, EBSCO, Web of Science, the Cochrane Central Register of Controlled Trials (CENTRAL), medRxiv, Scopus Preprints, and SSRN. Additionally, the reference lists of identified articles were manually reviewed. The detailed search strategy can be found in Supplementary Table S1 (available in the online version only). The first author conducted an initial screening of titles, abstracts, and full texts to assess their relevance and eligibility for inclusion in this paper. This process was collaboratively reviewed by the other authors, ensuring a thorough and systematic approach to the research.

### Study Selection

The retrieved studies were initially screened based on the relevance of their titles and abstracts. Articles were included if they reported outcomes using either MNT or CFNG techniques. Studies in non-English languages and those involving animal subjects were excluded. Additionally, case series, case reports, literature reviews, and previous meta-analyses were not considered for inclusion. There were no restrictions on the date of publication. The PICO (Participants, Intervention, Comparison, and Outcomes) criteria used for this review are detailed in Table 1 . This systematic review adhered to the 2020 Preferred Reporting Items for Systematic Reviews and Meta-Analyses (PRISMA) guidelines, and the study protocol was preregistered in the International Prospective Register of Systematic Reviews (PROSPERO) database (ID: CRD42024576631). A search of PROSPERO confirmed that no other protocols addressing this topic had been registered.

**Table 1:** PICO criteria for the systematic review

Participants	Patients with facial nerve paralysis, both complete/incomplete and bilateral/unilateral.
Intervention	Masseteric nerve transposition
Comparison	Cross-facial nerve graft
Outcomes	Commissural excursion Commissural contraction velocity Recovery percentage Static lip symmetry • MNT versus CFNG: Static lip angle symmetry (in degrees) • MNT versus CFNG: Static lip length symmetry (in mm) Dynamic lip symmetry • MNT versus CFNG: Dynamic lip angle symmetry (in degrees) • MNT versus CFNG: Dynamic lip length symmetry (in mm) Total smile improvement Quality of life Satisfaction

Abbreviations: CFNG, cross-facial nerve graft; MNT, masseteric nerve transposition.

### Data Collection and Quality Assessment

Full-text articles meeting the eligibility criteria were independently retrieved and evaluated based on predefined selection parameters. Data from the included studies were carefully extracted and organized in Excel spreadsheets to ensure accuracy and completeness. This process was conducted by two authors (I.L.P. and R.P.), and any discrepancies were resolved through discussion until consensus was reached. The following baseline information was extracted from each study: “author name,” “publication year,” “study design,” “type of centre,” and “surgical approach.” Cohort characteristics such as patient demographics (sample size, age, sex, cause of paralysis, and denervation time) were also recorded. Data on continuous variables, including postoperative outcomes such as commissural excursion (CE), commissural contraction velocity (CCV), static and dynamic lip symmetry, and overall smile improvement on the reanimated side, were collected and expressed as means and standard deviations. For postoperative complications, dichotomous data were extracted, and qualitative analysis would have been performed if the data were heterogeneous.

Each study's methodological quality was independently assessed by two authors (K.E.S.S. and C.D.K.W.) using the Newcastle–Ottawa Scale (NOS). 33 Any disagreements were resolved through discussion or, if necessary, by consulting a third author (I.F.A.). Studies were rated as very good quality (7–9 stars), good quality (5–6 stars), satisfactory quality (3–4 stars), or unsatisfactory quality (0–2 stars).

### Statistical Analysis

Any relevant results from research that directly compare MNT and CFNG approaches will be subjected to a pairwise meta-analysis. Mean differences (MDs) will be utilized for continuous variables. Meta-analysis results will be presented in forest plots, with summary estimates, 95% confidence intervals (CIs), and relative study weights indicated by the midpoint, horizontal line, and square size, respectively. A diamond shape's midpoint and width, representing the mean and 95% CI, will serve as the overall summary statistic. When data are insufficient for meta-analysis, pooled summary cohort characteristics for each outcome of interest will be reported for both operative techniques. Statistical heterogeneity between studies will be evaluated using the *I* ^2^ statistic, with values above 50% suggesting substantial heterogeneity. In this analysis, the effects model is chosen depending on the similarity of the data, including factors such as tools, units of measurement, and the presence of heterogeneity. All statistical analyses will be performed employing Review Manager version 5.4.1 (Cochrane Collaboration, Software Update, Oxford, United Kingdom).

## Results

### Study Characteristics

**Fig. 1:**
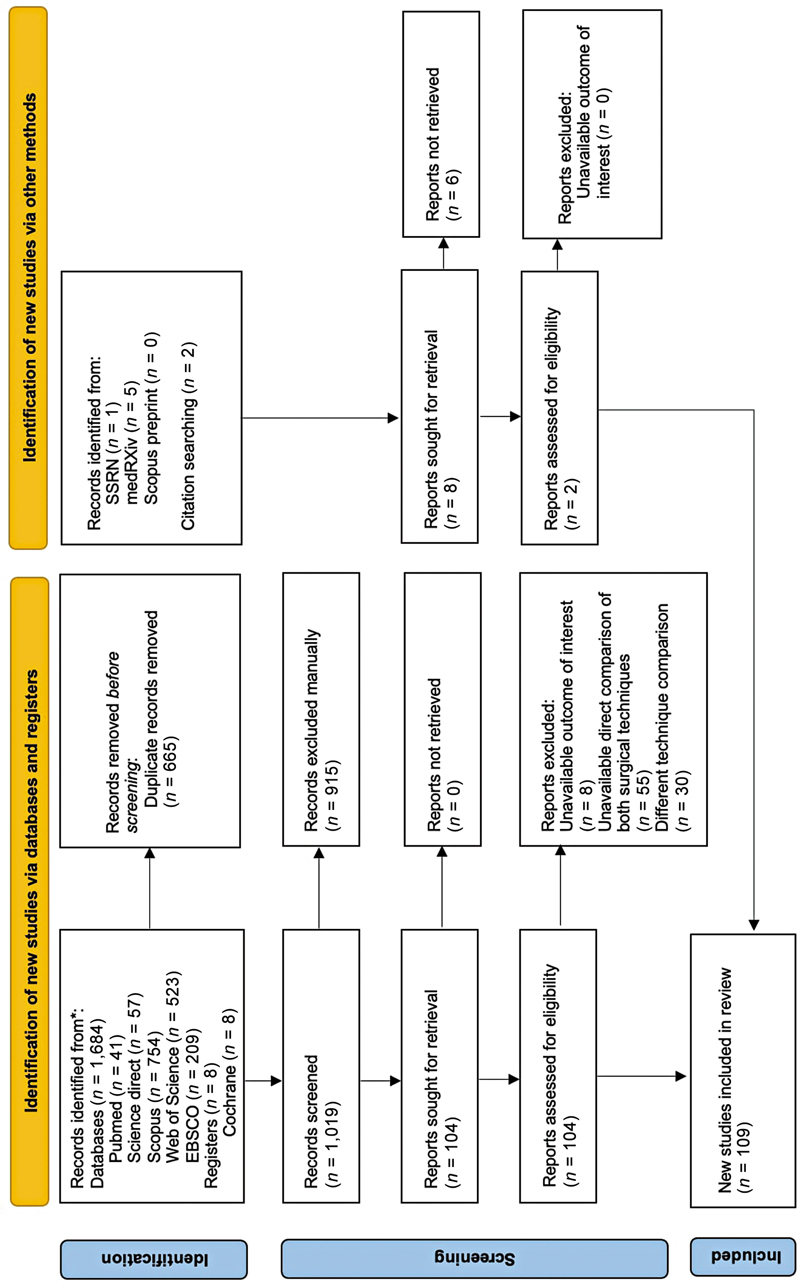
PRISMA flow diagram data added to the PRISMA template (from Page MJ, McKenzie JE, Bossuyt PM, Boutron I, Hoffmann TC, Mulrow CD, et al. The PRISMA 2020 statement: An updated guideline for reporting systematic reviews. BMJ. 2021;372: n71) under the terms of the Creative Commons Attribution License. PRISMA, Preferred Reporting Items for Systematic Reviews and Meta-Analyses. *Consider, if feasible to do so, reporting the number of records identified from each database or register searched (rather than the total number across all databases/registers).

A total of 1,684 articles were identified through the literature search, with 665 duplicates being removed. After an initial screening of titles and abstracts, 104 articles were deemed relevant and underwent a full-text evaluation based on the established inclusion and exclusion criteria. Following this assessment, seven articles met the criteria for inclusion in the quantitative analysis. Additionally, two more eligible articles were identified through screening the reference lists of the included studies. Ultimately, 11 studies were included in the final quantitative and qualitative synthesis ( Fig. 1 ). All of the included studies were retrospective cohort studies ( *n* = 11), with the majority conducted in the United States ( *n* = 5). The remaining studies were from Spain ( *n* = 2), Canada ( *n* = 2), India ( *n* = 1), and China ( *n* = 1). All studies were conducted in single-center settings. The quality of the studies was rated as good ( *n* = 2) to very good ( *n* = 9; Supplementary Table S2 [available in the online version only]).

### Cohort Description

The studies included a total of 569 patients, of whom 173 underwent MNT procedures. The age range of patients was broad, from 4 to 80 years. Nine studies reported sex distribution, with 41.5% (267/643) of the patients being male. In most studies ( *n* = 6), tumors were the primary cause of facial paralysis, followed by other etiologies such as Bell's palsy ( *n* = 3), congenital paralysis ( *n* = 1), and trauma ( *n* = 1). Five studies reported cases of long-standing paralysis, with a denervation time exceeding 24 months. Complete paralysis was predominant in most studies ( *n* = 8), while three studies reported cases of incomplete paralysis. The outcomes analyzed across the studies were as follows: CE ( *n* = 9), CCV ( *n* = 2), recovery percentage ( *n* = 2), static lip symmetry ( *n* = 2), dynamic lip symmetry ( *n* = 2), and total smile improvement ( *n* = 2; Table 2 ).

**Table 2:** Demographic data and sample characteristics

Number	Study, year, country	Title	Single/Multicenter	Study design	Surgical technique		Patient population				Denervation time	Follow-up (months)	Outcome	Evaluation tools
					Intervention	Control	*n* a	Age (years)	Sex	Cause				
1	Hontanilla et al (2018), Spain 37	Cross-face nerve grafting versus masseteric-to- facial nerve transposition for reanimation of incomplete facial paralysis: A comparative study using the FACIAL CLIMA evaluating system.	Single center	Cohort retrospective	MNT	CFNG	28	MNT (16–43) CFNG (19–58)	**M:F** MNT (5:13) CFNG (3:7)	The main cause was **Bell's palsy**	MNT (23–60) CFNG (26–56) months	MNT (25–44) CFNG (24–47) months	CD (in mm), CCV (in mm/s), satisfaction %, spontaneous %	FACIAL CLIMA software
2	Hontanilla et al (2013), Spain 1	Facial reanimation with gracilis muscle transfer neurotised to cross-facial nerve graft versus masseteric nerve: A comparative study using the FACIAL CLIMA evaluating system.	Single center	Cohort retrospective	FGMT to CFNG	FGMT to MNT	47	MNT (40.7 ± 13.8) CFNG (42.4 ± 10.1)	MNT (12:15) CFNG (7:13)	The main cause was **acoustic neurinoma**	MNT (108.4 ± 152.4) CFNG (121.7 ± 157.8) months	MNT (33.2 ± 12.2) CFNG (38.4 ± 15.7)	CD (in mm), CCV (in mm/s)	FACIAL CLIMA software
3	Alison K. Snyder-Warwick et al (2015), Canada 54	The degree of facial movement following microvascular muscle transfer in pediatric facial reanimation depends on donor motor nerve axonal density.	Multicenter	Cohort retrospective	FGMT to MNT	FGMT to CFNG	34	MNT (9.5) CFNG (10.2)	–	–	–	–	Commissural excursion (in mm), average myelinated axons	SMILE software
4	Kalra et al (2022), India 38	Facial reanimation using free functional muscle transfer: Lessons learnt from a long-term experience comparing innervation with cross facial nerve graft and masseter nerve.	Single center	Cohort retrospective	FFMT to CFNG	FFMT to MNT	205	4–62 years	93:112	The main cause was **Bell's palsy**	(1–48) years	–	Commissural excursion (in mm), satisfaction %, desc acute complication	–
5	Liang et al (2024), China 48	Comparison of outcomes of facial reanimation between the use of cross-facial nerve graft and the masseteric nerve as the donor nerve for reinnervation of gracilis muscle flap transfer.	Single center	Cohort retrospective	FGMT to MNT	FGMT to CFNG	21	MNT (48.9 ± 6.2) CFNG (19.6 ± 4.6)	MNT (6:4) CFNG (5:6)	The main cause was **trauma**	MNT (5.7 ± 2.8) CFNG (11.3 ± 6.1) months	MNT (24.1 ± 12.4) CFNG (37.1 ± 18.3)	Commissural excursion (mm), static and dynamic lip symmetry angle (degrees)	Standardized lateral photographs after surgery
6	Bhama et al (2014), United States 50	Objective outcomes analysis following microvascular gracilis transfer for facial reanimation a review of 10 years' experience.	Single center	Cohort retrospective	GFTT to CFNG	GGFT to MNT	78	35 ± 18 (6–80)	52:72	The main cause was **tumors (intracranial neoplasm and vestibular schwannoma)**	128 ± 183	–	Commissural excursion (mm), static and dynamic lip symmetry angle (degrees and mm)	FACE Gram
7	Lindsay et al (2014), United States 34	The success of free gracilis muscle transfer to restore smile in patients with nonflaccid facial paralysis.	Single center	Cohort retrospective	Gracilis to MNT	Gracilis to CFNG	20	37.75 ± 15.93	04.16	The main cause was **vestibular schwannoma and Bell's palsy**	–	–	Commissural excursion (mm), static and dynamic lip symmetry angle (degrees and mm), FaCE score determining quality of life (synkinesis)	FACE Gram/FGS, FaCE score
8	Bae et al (2006), Canada 30	A comparison of commissure excursion following gracilis muscle transplantation for facial paralysis using a cross-face nerve graft versus the motor nerve to the masseter nerve.	Single center	Cohort retrospective	FFMT to CFNG	FFMT to MNT	36	Mean age: MNT (8.7) CFNG (9.9) years	MNT (21:29) CFNG (29:41)	The main cause was **congenital**	Long-standing (congenital)	–	Operation time (hours), circumference of muscle (%), lengths of muscle used (cm), commissural excursion	Measurement using standardized photographs
9	Lindsay et al (2014), United States 35	Quality-of-life improvement after free gracilis muscle transfer for smile restoration in patients with facial paralysis.	Single center	Cohort retrospective	FGMT to MNT	FGMT to CFNG	72	40.1 ± 16.7 (20 patients; <14 years)	25;41	The main cause was **brain tumors**	–	–	FaCE score determining Quality of Life (synkinesis)	FaCE Score
10	Faris C. et al (2017), United States 55	Free-gracilis muscle transfer for smile reanimation after treatment for advanced parotid malignancy.	Single center	Cohort retrospective	MNT	CFNG	11	27–67	5;7	The main cause was **tumors**	12–204 months	–	Commissural excursion (in mm)	FACE, SaCE score
11	Hadlock et al (2011), United States 36	Free gracilis transfer for smile in children: The Massachusetts Eye and Ear Infirmary experience in excursion and quality-of-life changes.	Single center	Cohort retrospective	MNT (trigeminal)	CFNG	17	4–18 years	?	The main cause was **brain tumors**	–	–	FaCE score determining Quality of Life (synkinesis)	FaCE score

Abbreviations: CFNG, cross-facial nerve graft; FGMT, free gracilis muscle transfer; MNT, masseteric nerve transposition; GFTT, gracilis free tissue transfer; FFMT, free functional muscle transfer.

a *n* stands for total patients.

### Meta-Analysis

#### Primary Outcomes

##### Commissural Excursion

**Fig. 2:**
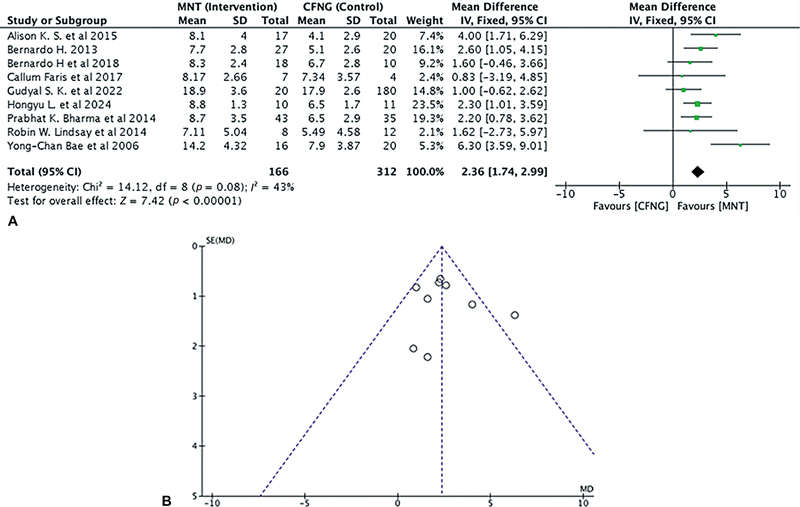
( **A** ) Forest plot of the commissural excursion in the MNT versus the CFNG procedure. ( **B** ) Funnel plot of the commissural excursion in the MNT versus the CFNG procedure. CFNG, cross-facial nerve graft; CI, confidence interval; MD, mean difference; MNT, masseteric nerve transposition; SD, standard deviation.

The smile excursion results were summarized in Table 2 . Nine studies ( *n* = 478) compared CE in facial reanimation using MNT versus CFNG as the donor nerve. MNT was shown to have a considerably higher CE, indicating more similarity to the healthy score (if the disparities between the two sides were ignored, the most symmetrical smile would be taken into consideration). There was no heterogeneity between the studies with *I* ^2^ < 50%. The analysis showed significantly different outcomes between MNT versus CFNG in the CE (in mm; pooled MD = 2.36, 95% CI = 1.74–2.99, *p* < 0.00001; Fig. 2 ).

##### Commissural Contraction Velocity

**Fig. 3:**
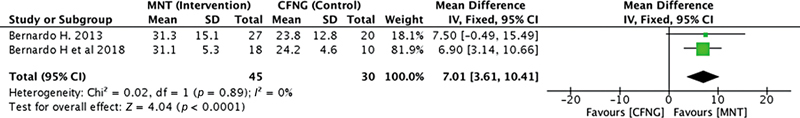
The commissural contraction velocity in the MNT versus the CFNG procedure. CFNG, cross-facial nerve graft; CI, confidence interval; MNT, masseteric nerve transposition; SD, standard deviation.

Two studies ( *n* = 75) compared CCV in facial reanimation using MNT versus CFNG as the donor nerve. MNT was shown to have considerably higher CCV than CFNG, indicating more similarity to the healthy score (if the disparities between the two sides were ignored, the most symmetrical smile would be taken into consideration). There was no heterogeneity between the studies with *I* ^2^ <50%. The analysis showed a significant difference between MNT and CFNG (pooled MD = 7.01, 95% CI = 3.61–10.41, *p* < 0.0001; Fig. 3 ).

##### Recovery Percentage

**Fig. 4:**
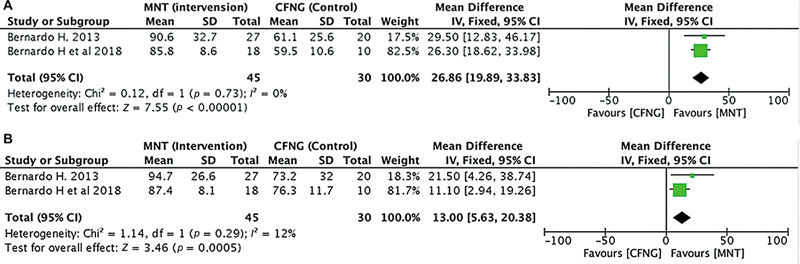
( **A** ) Recovery percentage of the commissural excursion in the MNT versus the CFNG procedure. ( **B** ) Recovery percentage of the commissural contraction velocity in the MNT versus the CFNG procedure. CFNG, cross-facial nerve graft; CI, confidence interval; MNT, masseteric nerve transposition; SD, standard deviation.

Two studies ( *n* = 75) compared recovery percentages in facial reanimation using MNT versus CFNG as the donor nerve. It was found that MNT had a much higher percentage of CCV than CFNG. A higher percentage (up to 100%) means the technique is more effective and closer to the healthy side score. There was no heterogeneity between the studies with *I* ^2^ < 50%. The study of the MNT recovery rate in CE showed a much better result than in CFNG (pooled MD = 26.86, 95% CI = 19.89–33.83, *p* < 0.00001; Fig. 4A ). Similarly, the MNT group showed a significantly higher recovery percentage in CCV (pooled MD = 13.00, 95% CI = 5.63–20.38, *p* = 0.0005; Fig. 4B ).

##### Static Lip Symmetry

**Fig. 5:**
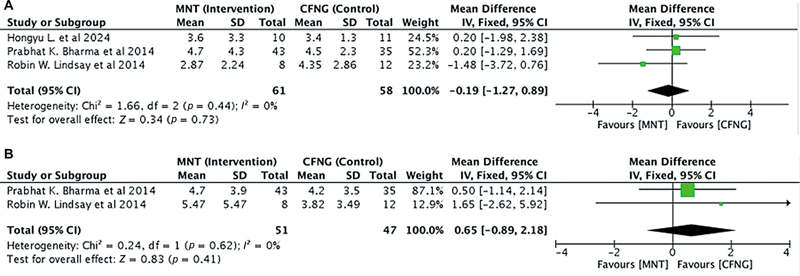
( **A** ) Static lip angle symmetry in the MNT versus the CFNG procedure. ( **B** ) Static lip length symmetry in the MNT versus the CFNG procedure. CFNG, cross-facial nerve graft; CI, confidence interval; MNT, masseteric nerve transposition; SD, standard deviation.

A total of 119 people ( *n* = 119) from all three studies were looked at to see how the static lip angle symmetry changed when MNT or CFNG nerves were used as the donor. The angle symmetry was measured between the vertical midline of the lower lip and the oral commissure. There was no heterogeneity between the studies with *I* ^2^ < 50% ( Fig. 5A ). The analysis showed non-significant association between MNT and static lip symmetry angle (pooled MD = −0.19, 95% CI = −1.27–0.89, *p* = 0.73; Fig. 5A ). When comparing lip length symmetry (in mm), we obtained two studies ( *n* = 98) Similarly, the MNT group showed no significant difference between the two interventions in static lip symmetry length (in mm; pooled MD = 0.65, 95% CI = −0.89–2.18, *p* = 0.41); there was no heterogeneity between the studies with *I* ^2^ < 50% ( Fig. 5B ).

##### Dynamic Lip Symmetry

**Fig. 6:**
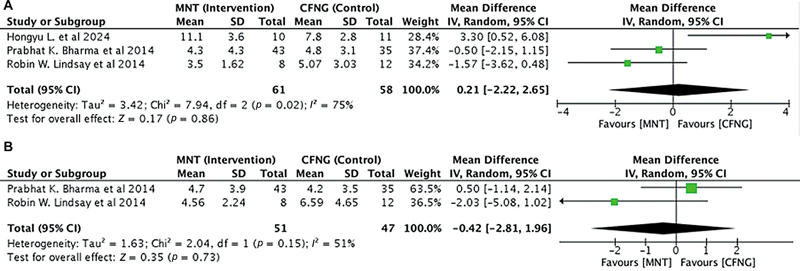
( **A** ) Dynamic lip angle symmetry in the MNT versus the CFNG procedure. ( **B** ) Dynamic lip length symmetry in the MNT versus the CFNG procedure. CFNG, cross-facial nerve graft; CI, confidence interval; MNT, masseteric nerve transposition; SD, standard deviation.

Three studies with 119 people ( *n* = 119) looked at the dynamic lip angle symmetry (in degrees) between the vertical midline of the lower lip and the oral commissure. The donor nerves used in these studies were either MNT or CFNG. There was heterogeneity between the studies with *I* ^2^ > 50% ( Fig. 5A ). The analysis showed no significant difference between MNT and dynamic lip angle symmetry (pooled MD = 0.21, 95% CI = −2.22–2.65, *p* = 0.86; Fig. 6A ). When comparing dynamic lip length symmetry (in mm), we obtained two studies ( *n* = 98). Similarly, the MNT group showed no significant difference between MNT versus CFNG in dynamic lip symmetry length (in mm; pooled MD = −0.42, 95% CI = −2.81–1.96, *p* = 0.73). There was heterogeneity between the studies with *I* ^2^ > 50% ( Fig. 6B ).

##### Total Smile Improvement

**Fig. 7:**
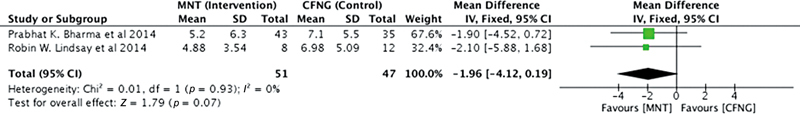
Total smile improvement in the MNT versus the CFNG procedure. CFNG, cross-facial nerve graft; CI, confidence interval; MNT, masseteric nerve transposition; SD, standard deviation.

Two studies ( *n* = 98) were included to compare smile improvement in facial reanimation using MNT versus CFNG as the donor nerve. MNT was shown to have considerably higher improvement than CFNG. There was no heterogeneity between the studies with *I* ^2^ < 50%. The analysis showed no significant difference between MNT and total smile improvement (pooled MD = −1.96, 95% CI −4.12–0.19, *p* = 0.07; Fig. 7 ).

#### Secondary Outcomes

##### Quality of Life

Three retrospective studies ( *n* = 99) assessed improvements in quality of life (QOL) following facial reanimation using the Face Clinimetric Evaluation (FaCE) instrument. 34 35 36 Patients who underwent free gracilis muscle transfer (FGMT) innervated by CFNG showed no statistically significant difference in FaCE score improvements compared with the MNT group. 35 In one study, 13 pediatric patients completed FaCE surveys, with preintervention and postintervention scores improving from 51.3 to 65.7, reflecting a statistically significant improvement. The average FaCE score improvement was 12.9 for CFNG and 17.7 for MNT. 36 Based on these studies, all three suggested that the QOL associated with the MNT procedure was generally higher than with CFNG, although two studies did not reach statistical significance. Thus, it can be concluded that MNT tends to be more favorable than CFNG in improving QOL.

##### Satisfaction

Two studies ( *n* = 233) reported patient satisfaction scores following reanimation surgery. 37 38 The masseter nerve gracilis transfer demonstrated favorable outcomes within the briefest recovery timeframe. In the CFNG group, five patients (2.8%, 3.1 ± 0.64) were unsatisfied and reported unsuccessful outcomes, while in the MNT group, one patient (5%, 3.45 ± 0.81) experienced failed results. 38 Another study by Hontanilla et al 37 reported that 16 patients (88.8%) were satisfied with MNT, while 8 patients (80%) were satisfied in the CFNG group. These studies suggest that satisfaction rates are generally higher with MNT compared with CFNG, with a lower incidence of dissatisfaction among MNT patients. However, one study noted a relatively higher rate of dissatisfaction in the MNT cohort, which may be due to differences in preoperative complexity.

## Discussion

Facial nerve paralysis is a severe disorder marked by partial or full loss of facial nerve function, resulting in facial asymmetry, deformity, and functional impairment. 19 One of the most important decisions in managing this condition is deciding which motor nerve to supply the paralyzed side. 19 In bilateral paralysis cases, where both facial nerves lack functionality, alternative cranial nerves have been evaluated for reinnervation. 30 Recently, MNT has gained prominence as the preferred technique for facial reanimation. 8 16 22 MNT uses the masseter nerve's descending branch while leaving the proximal branches intact, preventing masseter muscle atrophy. 39

The masseter nerve, the largest pure motor branch of the trigeminal nerve, 30 innervates the masseter muscle, which plays a crucial role in mastication. Its consistent anatomical structure, sufficient length, and proximity to the facial nerve branches 39 make it an ideal motor source for facial reanimation surgery. 11 23 24 39 The motor branch innervating the masseter muscle lies on the muscle's undersurface, generally extending vertically downward along the posterior margin, positioned just beneath the zygomatic arch. The nerve is found approximately 11 mm below the zygomatic arch, 38 mm anterior to the tragus, and 13 mm deep within the masseter muscle. 4 19 40 Prior research indicates that the mean distance from the coronoid notch to the entrance of the masseter nerve is 32 mm. 39 During surgery, the masseter nerve is carefully dissected to achieve the necessary length for reinnervation. 38

In contrast to MNT, the CFNG procedure involves connecting the motor branch of the facial nerve from the healthy, non-paralyzed side to a sural nerve graft. This technique carries the inherent risk of compromising facial nerve function on the unaffected side. 41 Additionally, CFNG is typically a multistage procedure, which increases the risk of failure. 14 The facial nerve's primary role is to provide motor innervation to the muscles responsible for facial expression. To identify the facial nerve branches on the paralyzed side, a periauricular incision is made, extending toward the modiolus until the desired branch is located, 1 usually found at the anterior border of the parotid gland. 42 The buccal or zygomatic branches are often chosen for coaptation with the cross-face sural nerve graft, although they can be challenging to distinguish anatomically. 30

The surgical durations of both MNT and CFNG vary depending on their complexity and the surgeon's expertise. Data from previous studies show that the CFNG procedure takes approximately 80 to 90 minutes for the first stage and 150 to 180 minutes for the second stage. In contrast, the MNT procedure, performed in a single stage, typically requires 170 to 200 minutes. This suggests that MNT, while taking longer in a single session, may be more time-efficient overall compared with the multistage CFNG. 38 However, no significant differences were observed in total operative time when comparing the muscle transplantation component of both techniques. 30

In a study by Snyder-Warwick et al, the masseteric nerve demonstrated an average of 5,289 myelinated fibers per square millimeter, compared with the 1,647 axons per square millimeter found in the CFNG, representing a 76% reduction in fiber density from the donor facial nerve. Neurotization using the masseteric nerve has proven to be a reliable technique for smile reanimation, producing robust, symmetrical, 28 and natural smiles with significant functional recovery. 18 These findings are in-line with our comprehensive comparison of both interventions, as summarized in this report ( Table 3 ).

**Table 3:** Comprehensive summary of outcomes derived from the forest plot analysis

Outcomes	Pooled MD (95% CI)	*I* ^2^	*p* -Value
Commissural excursion	2.36 (1.74–2.99)	43%	<0.00001
Commissural contraction velocity	7.01 (3.61–10.41)	0%	<0.0001
Recovery percentage • CE recovery percentage • CCV recovery percentage	26.86 (19.89–33.83) 13.00 (5.63–20.38)	0% 12%	<0.00001 0.0005
Static lip symmetry • By angle • By length	−0.19 (−1.27–0.89) 0.65 (−0.89–2.18)	0% 0%	0.73 0.41
Dynamic lip symmetry • By angle • By length	0.21 (−2.22–2.65) −0.42 (−2.81–1.96)	75% 51%	0.86 0.73
Total smile improvement	−1.96 (−4.12–0.19)	0%	0.07

Abbreviations: CCV, commissural contraction velocity; CE, commissural excursion; CI, confidence interval; MD, mean difference.

Previous research indicates that smile asymmetry is often a primary concern for patients with facial paralysis, as symmetry is a crucial determinant of facial attractiveness. The masseter nerve transfer has been shown to improve both facial symmetry and oral commissure excursion 22 due to its strong motor input. 13 Studies by Bianchi et al have established that the normal range of commissural movement is between 7 and 22 mm, with a mean of 14 mm. 43 Clinical observations suggest that muscles innervated by the masseter nerve exhibit greater excursion 16 44 compared with those utilizing CFNG. For instance, Roy et al reported that the masseter nerve transfer resulted in a greater smile excursion (10.0 mm) than CFNG (6.8 mm). 26 Additionally, quantitative assessments using tools such as FACEGram 12 35 and FACIAL CLIMA 31 have shown that over 75% of patients undergoing masseter nerve transfer experience significant improvements in commissural excursion and velocity. 22 This finding supports our current study, which demonstrates the advantages of masseter nerve transfer in enhancing CE. Notably, the oral commissure movement on the side treated with CFNG was significantly less than that on the healthy side and the masseter nerve transfer group. However, some studies suggest that the CFNG procedure may facilitate a degree of spontaneous movement that is not achievable with the masseter nerve.

CCV is another important parameter for assessing facial symmetry 45 and is evaluated alongside CE 17 42 46 in tools like FACIAL CLIMA. 31 A recent study by Hontanilla et al (2018) compared MNT and CFNG, reporting mean CCV values of 35.0 ± 4.5 for MNT and 33.6 ± 5.4 for CFNG. 37 A closer alignment of the CCV on the reanimated side to that of the healthy side indicates a higher likelihood of surgical success. Our analysis corroborates this, revealing that the CCV on the reanimated side following MNT is statistically significantly higher than that observed in the CFNG group. Furthermore, in a separate analysis of CCV, the healthy side and the reanimated side using masseter-innervated nerve transfer showed no significant difference (23.8 vs. 31.3, *p* = 0.29). This suggests that successful outcomes are associated with the reanimated side achieving results that closely resemble those of the healthy side.

CE and CCV values were converted into recovery percentages to evaluate the improvement rates of each technique based on the paralyzed side's recovery relative to the healthy side. 22 42 This study revealed that the MNT group exhibited significantly higher recovery rates for both commissural displacement and contraction velocity. An optimal smile is characterized by minimal differences between the reanimated and healthy sides; conversely, substantial discrepancies indicate asymmetrical movement.

Achieving symmetry in both static and dynamic facial expressions poses a considerable challenge in the reconstruction of facial nerve paralysis. Changes in oral commissural symmetry can be assessed through both horizontal and vertical dimensions, which collectively define the angle. A previous study demonstrated that gracilis free muscle transfer, powered by the masseteric nerve, led to significant improvements in horizontal length symmetry during both rest and smiling. 47 Symmetry was quantified by the ratio of oral commissure movement on the affected side compared with the healthy side, with significant enhancements noted during smiling. 12 Patients in the MNT group exhibited more substantial enhancements in static angle symmetry compared with those in the CFNG group. Furthermore, comparisons of dynamic angle differences in both groups indicated impressive improvements, with the masseter-innervated group achieving a larger angle. This group also demonstrated superior improvements in angle symmetry during both rest and smiling. 47 It has been suggested that a satisfactory smile is indicated when postoperative static and dynamic angles approach 90 degrees, and the smile excursion on the affected side closely approximates that of the healthy side. 48 However, it is unfortunate that the comparison of the two procedures in terms of static and dynamic lip symmetry did not reach statistical significance. This lack of significance may be attributed to various factors, including the patient's condition (unilateral or bilateral abnormalities), denervation time, 9 and the high level of dependence on the operator, who plays a critical role in determining the expected outcomes.

Facial paralysis is a serious condition that adversely affects patients' QoL and is linked to depression, making it an important issue in facial reanimation surgery. 2 49 Patient-reported outcome measures, including the Facial Clinimetric Evaluation (FaCE) Scale, offer clinically significant insights into QoL enhancements and have been widely employed in the literature. 35 50 The FaCE Scale serves multiple important clinical purposes, including assessing the natural progression of facial disability from the acute phase of paralysis through various recovery stages caused by different underlying factors. This tool enhances our understanding of patients' needs and their responses to various treatment modalities. Furthermore, the FaCE Scale complements clinician-administered instruments designed to quantify facial impairment. 25 51

In this study, patients who underwent the CFNG procedure did not show a statistically significant difference in FaCE scores compared with those who received MNT. One potential explanation for this finding is that the mean age of the MNT group was significantly higher than that of the CFNG group, 35 which is relevant since age can influence axonal density. 14 CFNG is considered an ideal choice for those patients who have a sufficiently strong contralateral facial nerve. 19 Previous research indicates that younger patients with severe facial paralysis tend to benefit more from early MNT, particularly in cases of traumatic injury. 25 However, other studies have suggested that patient age at the time of surgery does not significantly impact aesthetic or functional outcomes. 52

This is an important aspect to consider the possibility of complication when comparing different techniques of facial reanimation. In fact, from previous studies, complication rates were not significantly different between groups. Some mild to severe complications might happen postoperatively and during the healing phase. These complications vary, including postoperative bleeding, hematoma, wound infections, the risk of wound dehiscence, muscle atrophy, and flap failure. 4 47 53 In the CFNG procedure, there is always the risk of damage to the function of the facial nerve, weakness of the smile on the nonparalyzed side, once the branch of the zygomatic or buccal nerve is transected for anastomosis. There will be numbness on the lateral aspect of the foot from the sural nerve harvest. If the deep peroneal nerve is accidentally transected, foot drop remains a possibility. 41 Whereas in the MNT procedure, minor synkinesis was also observed in the masseter nerve transfer group. 44 A previous study stated that there have been no reports of donor site morbidity related to loss of masseter muscle function and masseter muscle atrophy. 4 Although there is a possibility of various complications occurring, with good postoperative care, these complaints can be resolved, and rehabilitation is very important to begin around 6 weeks postoperatively, including massage, smile training or induction exercise, and muscle stimulation. Smile training in front of the mirror is quite important. 38

This study has several limitations, including a small sample size and an unbalanced distribution of outcome parameters, which may affect the statistical significance of the results. This meta-analysis includes two studies 34 35 with a potential overlap in patient cohorts, representing a minor limitation concerning the independence of data points. Nonetheless, the impact is anticipated to be minimal. Additionally, conducting head-to-head comparisons proved challenging during data collection with the predominance of retrospective studies, resulting in potential bias and limited ability to access broader outcomes such as axonal improvement and spontaneous smiles. Therefore, further comparative studies with larger sample sizes are necessary to provide more robust evidence regarding the benefits of the MNT procedure. Future research is expected to address the gaps identified in this review, particularly in areas where the findings were non-significant, by including a diverse patient population and employing standardized outcome measures to strengthen the evidence supporting the efficacy of MNT in facial reanimation surgery. Additionally, outcomes should be evaluated using reliable and standardized assessment parameters to ensure the validity of future studies.

## Conclusion

In conclusion, various facial nerve reconstruction techniques are viable options for facial reanimation, considering the individual patient's circumstances and preoperative conditions. This meta-analysis highlights the significant advantages of MNT over CFNG procedures. Improvements in lip movement and smile functionality, as measured by critical parameters such as CE, CCV, and both static and dynamic symmetry, demonstrate that MNT is a highly preferred approach for facial reanimation. Additionally, secondary outcomes suggest that MNT may further benefit facial reanimation. However, more comprehensive data are needed to analyze these secondary outcomes in-depth.

## References

[1] Hontanilla B Marre D Cabello Á Facial reanimation with gracilis muscle transfer neurotized to cross-facial nerve graft versus masseteric nerve: a comparative study using the FACIAL CLIMA evaluating system Plast Reconstr Surg 2013 131 06 1241 1252 23416438 10.1097/PRS.0b013e31828bd4da

[2] Özücer B Çam O H Effect of distal masseter to facial nerve transfer in paralytic patients with preserved facial nerve continuity on improving Scaled Measurement of Improvement in Lip Excursion (SMILE): A vectoral analysis Turk Arch Otorhinolaryngol 2020 58 04 249 253 33554200 10.5152/tao.2020.5823 PMC7846303

[3] Krishnan K G Schackert G Seifert V Outcomes of microneurovascular facial reanimation using masseteric innervation in patients with long-standing facial palsy resulting from cured brainstem lesions Neurosurgery 2010 67 03 663 674, discussion 674 20651635 10.1227/01.NEU.0000375531.77489.79

[4] Biglioli F Frigerio A Colombo V Masseteric-facial nerve anastomosis for early facial reanimation J Craniomaxillofac Surg 2012 40 02 149 155 21463951 10.1016/j.jcms.2011.03.005

[5] Sforza C Tarabbia F Mapelli A Facial reanimation with masseteric to facial nerve transfer: A three-dimensional longitudinal quantitative evaluation J Plast Reconstr Aesthet Surg 2014 67 10 1378 1386 24939829 10.1016/j.bjps.2014.05.039

[6] Hontanilla B Marre D Cabello A Masseteric nerve for reanimation of the smile in short-term facial paralysis Br J Oral Maxillofac Surg 2014 52 02 118 123 24148699 10.1016/j.bjoms.2013.09.017

[7] Yang S F Xie Y Kim J C Outcomes of facial symmetry and tone at rest after masseteric-to-facial nerve transfer Facial Plast Surg Aesthet Med 2021 23 05 357 361 32757958 10.1089/fpsam.2020.0312

[8] Owusu J A Truong L Kim J C Facial nerve reconstruction with concurrent masseteric nerve transfer and cable grafting JAMA Facial Plast Surg 2016 18 05 335 339 27197116 10.1001/jamafacial.2016.0345

[9] Kollar B Weiss J BW Kiefer J Eisenhardt S U Functional outcome of dual reinnervation with cross-facial nerve graft and masseteric nerve transfer for facial paralysis Plast Reconstr Surg 2024 153 1178e 1190e 10.1097/PRS.0000000000010888 37384874

[10] Lenz Y Kiefer J Dietrich F Stark G B Eisenhardt S U Pre-operative masseter muscle EMG activation during smile predicts synchronicity of smile development in facial palsy patients undergoing reanimation with the masseter nerve: A prospective cohort study ^✰^ J Plast Reconstr Aesthet Surg 2019 72 03 505 512 30509736 10.1016/j.bjps.2018.11.011

[11] van Veen M M Dusseldorp J R Quatela O Patient experience in nerve-to-masseter-driven smile reanimation J Plast Reconstr Aesthet Surg 2019 72 08 1265 1271 31060989 10.1016/j.bjps.2019.03.037

[12] Oh T S Kim H B Choi J W Jeong W S Facial reanimation with masseter nerve-innervated free gracilis muscle transfer in established facial palsy patients Arch Plast Surg 2019 46 02 122 128 30934175 10.5999/aps.2018.00717 PMC6446028

[13] Hontanilla B Cabello A Spontaneity of smile after facial paralysis rehabilitation when using a non-facial donor nerve J Craniomaxillofac Surg 2016 44 09 1305 1309 27460946 10.1016/j.jcms.2016.06.031

[14] Bayezid K C Joukal M Karabulut E Macek J Moravcová L Streit L Donor nerve selection in free gracilis muscle transfer for facial reanimation. A systematic review and meta-analysis of clinical outcomes J Plast Reconstr Aesthet Surg 2023 82 31 47 37148809 10.1016/j.bjps.2023.04.014

[15] Woo S H Kim Y C Kim J Kwon S Oh T S Artificial intelligence-based numerical analysis of the quality of facial reanimation: A comparative retrospective cohort study between one-stage dual innervation and single innervation J Craniomaxillofac Surg 2023 51 05 265 271 37353406 10.1016/j.jcms.2023.05.012

[16] Kannan R Y Neville C Gwynn T Young K Venables V Nduka C Evaluation of masseteric nerve-based selective neurotization for multivectorial augmentation of the weak smile JAMA Facial Plast Surg 2019 21 04 340 341 31046074 10.1001/jamafacial.2019.0035 PMC6499132

[17] Hontanilla B Marré D Comparison of hemihypoglossal nerve versus masseteric nerve transpositions in the rehabilitation of short-term facial paralysis using the FACIAL CLIMA evaluating system Plast Reconstr Surg 2012 130 05 662e 672e 10.1097/PRS.0b013e318267d5e8 23096620

[18] Tzafetta K Al-Hassani F Pinto-Lopes R Wade R G Ahmad Z Long-term outcomes of dual innervation in functional muscle transfers for facial palsy J Plast Reconstr Aesthet Surg 2021 74 10 2664 2673 33853750 10.1016/j.bjps.2021.03.007

[19] Bianchi B Copelli C Ferrari S Ferri A Bailleul C Sesenna E Facial animation with free-muscle transfer innervated by the masseter motor nerve in unilateral facial paralysis J Oral Maxillofac Surg 2010 68 07 1524 1529 20417006 10.1016/j.joms.2009.09.024

[20] Telich-Tarriba J E Orihuela-Rodríguez A Rivera-Priego A L Changes in electrical activity of the masseter muscle and masticatory force after the use of the masseter nerve as donor in facial reanimation surgery Indian J Plast Surg 2020 53 01 59 63 32425369 10.1055/s-0039-3400673 PMC7225014

[21] Wang W Yang C Li W Li Q Zhang Y Masseter-to-facial nerve transfer: Is it possible to rehabilitate the function of both the paralyzed eyelid and the oral commissure? Aesthetic Plast Surg 2012 36 06 1353 1360 23052376 10.1007/s00266-012-9951-9

[22] Hontanilla B Marre D Masseteric-facial nerve transposition for reanimation of the smile in incomplete facial paralysis Br J Oral Maxillofac Surg 2015 53 10 943 948 26143295 10.1016/j.bjoms.2015.06.011

[23] Lu G N Han R Lee E Byrne P Boahene K Predicting resting oral commissure tone outcomes following masseter nerve transfer in facial reanimation Facial Plast Surg Aesthet Med 2021 23 04 249 254 32985899 10.1089/fpsam.2020.0195

[24] Sforza C Frigerio A Mapelli A Facial movement before and after masseteric-facial nerves anastomosis: A three-dimensional optoelectronic pilot study J Craniomaxillofac Surg 2012 40 05 473 479 21872484 10.1016/j.jcms.2011.07.004

[25] Li T Liu Y Zhang S Yang W Zuo M Liu X Multiple model evaluation of the masseteric-to-facial nerve transfer for reanimation of the paralyzed face and quick prognostic prediction Front Surg 2022 9 735231 35372492 10.3389/fsurg.2022.735231 PMC8964509

[26] Roy M Corkum J P Shah P S Effectiveness and safety of the use of gracilis muscle for dynamic smile restoration in facial paralysis: A systematic review and meta-analysis J Plast Reconstr Aesthet Surg 2019 72 08 1254 1264 31204152 10.1016/j.bjps.2019.05.027

[27] Hontanilla B Olivas-Menayo J Marré D Cabello Á Aubá C Maximizing the smile symmetry in facial paralysis reconstruction: An algorithm based on twenty years' experience Facial Plast Surg 2021 37 03 360 369 34062562 10.1055/s-0041-1722905

[28] Jones R M Victor J D Conte M M Detecting symmetry and faces: Separating the tasks and identifying their interactions Atten Percept Psychophys 2012 74 05 988 1000 22419373 10.3758/s13414-012-0273-4 PMC3392169

[29] Biglioli F Colombo V Tarabbia F Recovery of emotional smiling function in free-flap facial reanimation J Oral Maxillofac Surg 2012 70 10 2413 2418 22310454 10.1016/j.joms.2011.11.031

[30] Bae Y C Zuker R M Manktelow R T Wade S A comparison of commissure excursion following gracilis muscle transplantation for facial paralysis using a cross-face nerve graft versus the motor nerve to the masseter nerve Plast Reconstr Surg 2006 117 07 2407 2413 16772949 10.1097/01.prs.0000218798.95027.21

[31] Hontanilla B Aubá C Automatic three-dimensional quantitative analysis for evaluation of facial movement J Plast Reconstr Aesthet Surg 2008 61 01 18 30 17569607 10.1016/j.bjps.2007.03.037

[32] Vincent A G Bevans S E Robitschek J M Wind G G Hohman M H Masseteric-to-facial nerve transfer and selective neurectomy for rehabilitation of the synkinetic smile JAMA Facial Plast Surg 2019 21 06 504 510 31465094 10.1001/jamafacial.2019.0689 PMC6902154

[33] Wells G A Shea B O'Connell D Peterson J Welch V Losos M Tugwell P The Newcastle-Ottawa Scale (NOS) for assessing the quality of nonrandomised studies in meta-analysesOttawa Hospital Reasearch Institute. Published May 3, 2021. Accessed August 13, 2024.https://www.ohri.ca/programs/clinical_epidemiology/oxford.asp.

[34] Lindsay R W Bhama P Weinberg J Hadlock T A The success of free gracilis muscle transfer to restore smile in patients with nonflaccid facial paralysis Ann Plast Surg 2014 73 02 177 182 24051452 10.1097/SAP.0b013e3182a0df04

[35] Lindsay R W Bhama P Hadlock T A Quality-of-life improvement after free gracilis muscle transfer for smile restoration in patients with facial paralysis JAMA Facial Plast Surg 2014 16 06 419 424 25275339 10.1001/jamafacial.2014.679

[36] Hadlock T A Malo J S Cheney M L Henstrom D K Free gracilis transfer for smile in children: The Massachusetts Eye and Ear Infirmary Experience in excursion and quality-of-life changes Arch Facial Plast Surg 2011 13 03 190 194 21576665 10.1001/archfacial.2011.29

[37] Hontanilla B Olivas J Cabello Á Marré D Cross-face nerve grafting versus masseteric-to-facial nerve transposition for reanimation of incomplete facial paralysis: A comparative study using the FACIAL CLIMA evaluating system Plast Reconstr Surg 2018 142 02 179e 191e 10.1097/PRS.0000000000004612 30045184

[38] Kalra G S Kalra S Gupta S Facial reanimation using free functional muscle transfer: lessons learnt from a long term experience comparing innervation with cross facial nerve graft and masseter nerve J Craniofac Surg 2022 33 08 e791 e796 35258013 10.1097/SCS.0000000000008606

[39] Park H Jeong S S Oh T S Masseter nerve-based facial palsy reconstruction Arch Craniofac Surg 2020 21 06 337 344 33663141 10.7181/acfs.2020.00682 PMC7933725

[40] Hontanilla B Qiu S S Transposition of the hemimasseteric muscle for dynamic rehabilitation of facial paralysis J Craniofac Surg 2012 23 01 203 205 22337408 10.1097/SCS.0b013e31824190a6

[41] Peng G L Azizzadeh B Cross-facial nerve grafting for facial reanimation Facial Plast Surg 2015 31 02 128 133 25958898 10.1055/s-0035-1549046

[42] Hontanilla B Marre D Cabello A Cross-face nerve grafting for reanimation of incomplete facial paralysis: Quantitative outcomes using the FACIAL CLIMA system and patient satisfaction J Reconstr Microsurg 2014 30 01 25 30 23818253 10.1055/s-0033-1349347

[43] Bianchi B Copelli C Ferrari S Ferri A Sesenna E Use of the masseter motor nerve in facial animation with free muscle transfer Br J Oral Maxillofac Surg 2012 50 07 650 653 21885172 10.1016/j.bjoms.2011.07.019

[44] Wang W J Zhu W D Tremp M Facial reanimation with interposition nerve graft or masseter nerve transfer: a comparative retrospective study Neural Regen Res 2022 17 05 1125 1130 34558541 10.4103/1673-5374.324862 PMC8552848

[45] Ueda K Harii K Yamada A Long-term follow-up of nerve conduction velocity in cross-face nerve grafting for the treatment of facial paralysis Plast Reconstr Surg 1994 93 06 1146 1149 8171133 10.1097/00006534-199405000-00006

[46] Hontanilla B Vila A Comparison of hemihypoglossal-facial nerve transposition with a cross-facial nerve graft and muscle transplant for the rehabilitation of facial paralysis using the FACIAL CLIMA method J Plast Surg Hand Surg 2012 46 01 25 31 22455573 10.3109/2000656X.2011.644716

[47] Oyer S L Nellis J Ishii L E Boahene K D Byrne P J Comparison of objective outcomes in dynamic lower facial reanimation with temporalis tendon and gracilis free muscle transfer JAMA Otolaryngol Head Neck Surg 2018 144 12 1162 1168 30325983 10.1001/jamaoto.2018.1964 PMC6583085

[48] Liang H Yang Z Ma N Wang W Li Y Comparison of outcomes of facial reanimation between the use of cross-facial nerve graft and the masseteric nerve as the donor nerve for reinnervation of gracilis muscle flap transfer J Craniofac Surg 2024 35 01 172 176 38294299 10.1097/SCS.0000000000009775

[49] Park J H Park S O Chang H Facial reanimation using free partial latissimus dorsi muscle transfer: Single versus dual innervation method J Craniomaxillofac Surg 2022 50 10 778 784 36257900 10.1016/j.jcms.2022.09.001

[50] Bhama P K Weinberg J S Lindsay R W Hohman M H Cheney M L Hadlock T A Objective outcomes analysis following microvascular gracilis transfer for facial reanimation: A review of 10 years' experience JAMA Facial Plast Surg 2014 16 02 85 92 24481538 10.1001/jamafacial.2013.2463

[51] Kahn J B Gliklich R E Boyev K P Stewart M G Metson R B McKenna M J Validation of a patient-graded instrument for facial nerve paralysis: The FaCE scale Laryngoscope 2001 111 03 387 398 11224766 10.1097/00005537-200103000-00005

[52] Liang H Chen S Yang Z Facial animation with free functional gracilis transfer innervated by the cross-facial nerve graft J Craniofac Surg 2021 32 05 1754 1757 33229991 10.1097/SCS.0000000000007251

[53] Murphey A W Clinkscales W B Oyer S L Masseteric nerve transfer for facial nerve paralysis a systematic review and meta-analysis JAMA Facial Plast Surg 2018 20 02 104 110 29222560 10.1001/jamafacial.2017.1780 PMC5885967

[54] Snyder-Warwick A K Fattah A Y Zive L Halliday W Borschel G H Zuker R M The degree of facial movement following microvascular muscle transfer in pediatric facial reanimation depends on donor motor nerve axonal density Plast Reconstr Surg 2015Feb;135 02 370e 381e 10.1097/PRS.0000000000000860 25626821

[55] Faris C Heiser A Hadlock T Jowett N Free gracilis muscle transfer for smile reanimation after treatment for advanced parotid malignancy Head Neck 2018Mar;40 03 561 568 29155463 10.1002/hed.25022

