# Supplementary material for: Postoperative Outcomes of Masseteric Nerve Transposition versus Cross-Facial Nerve Graft in Facial Reanimation: A Systematic Review and Meta-Analysis
Source: Arch Plast Surg. 2026 Jan 30;53(1):60–71. doi: 10.1055/a-2707-0408 (PMC12858318; doi:10.1055/a-2707-0408)
Supplement: Supplementary file 1 — Supplementary Material [file 10-1055-a-2707-0408-s24oct0170rev.pdf]

Supplementary Data

Supplementary Table 1. Detailed search strategies

Database	Search terms
PubMed	{((facial nerve paralysis [MeSH Terms]) OR (facial nerve paraly* [Title/Abstract]) OR (facial paralysis [Title/Abstract]) OR (facial paraly* [Title/Abstract]) OR (facial palsy [Title/Abstract]) OR (facial paresis [Title/Abstract]) OR (facial reanimation [Title/Abstract]) AND ((masseteric nerve transposition [MeSH Terms]) OR (masseteric nerve transfer [Title/Abstract]) OR (cross facial nerve graft [Title/Abstract]) OR (master nerve [Title/Abstract]) OR (nerve to masseter [Title/Abstract]) OR (masseteric nerve transposition* [Title/Abstract]))}
Cochrane	((MeSH descriptor: [facial paralysis]) OR ((facial paralysis):ti,ab,kw) OR ((facial paresis):ti,ab,kw) OR ((facial reanimation):ti,ab,kw) AND ((MeSH descriptor: [masseteric nerve transposition]) OR ((masseteric nerve transposition):ti,ab,kw) OR ((cross facial nerve graft):ti,ab,kw) OR ((masseteric nerve):ti,ab,kw) OR ((nerve to masseter):ti,ab,kw))
Web of Science	((TS=facial nerve paralysis) OR TS= (facial paralysis) OR TS=(facial palsy) OR TS=(facial paresis) OR TS=(facial reanimation)) AND ((TS=(masseteric nerve transposition) OR TS=(masseteric nerve transfer) OR TS=(cross facial nerve graft) OR TS=(facial reanimation))
EBSCO (CINAHL, Dentistry and Oral Medicine)	(AB “facial nerve paralysis” OR AB “facial palsy” OR AB “facial paresis” OR AB “facial reanimation”) AND (AB “masseteric nerve transposition”) OR AB “masseteric nerve transfer” OR AB “cross facial nerve graft” OR AB “masseter nerve” OR AB “nerve to masseter”)
Scopus	(TITLE-ABS-KEY (“facial nerve paralysis” OR “facial palsy” OR “facial paresis” OR “facial reanimation”)) AND (TITLE-ABS-KEY (“masseteric nerve transposition”) OR “masseteric nerve transfer” OR “cross facial nerve graft” OR “masseter nerve” OR “nerve to masseter”))
Science Direct	((facial nerve paralysis) OR (facial palsy) OR (facial paresis) OR (facial reanimation) AND ((masseteric nerve transposition) OR (masseteric nerve transfer) OR (cross facial nerve graft) OR (masseter nerve) OR (nerve to masseter))
MedxRiv	((facial nerve paralysis) OR (facial palsy) OR (facial paresis) OR (facial reanimation) AND (masseteric nerve transposition) OR (masseteric nerve transfer) OR (cross facial nerve graft) OR (masseter nerve) OR (nerve to masseter))
Scopus Preprint	(TITLE-ABS-KEY (“facial nerve paralysis” OR “facial palsy” OR “facial paresis” OR “facial reanimation”)) AND (TITLE-ABS-KEY (“masseteric nerve transposition”) OR “masseteric nerve transfer” OR “cross facial nerve graft” OR “masseter nerve” OR “nerve to masseter”))
SSRN	((facial nerve paralysis) OR (facial palsy) OR (facial paresis) OR (facial reanimation) AND (masseteric nerve transposition) OR (masseteric nerve transfer) OR (cross facial nerve graft) OR (masseter nerve) OR (nerve to masseter))

Supplementary Table 2. The Newcastle-Ottawa Scale (NOS) Quality Assessment.

	Selection				Comparability	Outcome				
Author	Representativeness of the exposed cohort	Selection of the non-exposed cohort	Ascertainment of exposure	Demonstration that outcome of interest was not present at start of study	Comparability of cohorts on the basis of the design or analysis	Assessment of outcome	Was follow-up long enough for outcomes to occur	Adequacy of follow up of cohorts	NOS score	Overall assessment
Benardo Hotanilla et al, 2018	1	1	1	1	1	1	1	1	8	Very good quality
Benardo Hotanilla et al, 2013	1	1	1	1	1	1	1	1	8	Very good quality
Alison K. Snyder-Warwick et al, 2015	1	1	1	0	0	1	1	1	6	Good quality
Gurdyal S. K et al, 2022	1	1	1	1	2	1	1	1	9	Very good quality
Hongyu Liang et al, 2024	1	1	1	1	1	1	1	1	8	Very good quality
Prabhat K. Bhama et al, 2014	1	1	1	1	1	1	1	1	8	Very good quality
Robin W. Lindsay et al, 2014	1	1	1	1	1	1	1	1	8	Very good quality
Yong-Chan Bae et al, 2006	1	1	1	1	2	1	1	1	9	Very good quality
Robin W. Lindsay et al, 2014	1	1	1	1	1	1	1	1	8	Very good quality
Callum F. et al, 2017	1	1	1	1	1	1	1	1	8	Very good quality
Tessa A. H. et al, 2011	1	1	1	1	0	1	1	0	6	Good quality
